# The comparison of two different weaning trial methods for patients with chronic obstructive lung disease (copd) in a cardiothoracic intensive care unit (icu)

**DOI:** 10.1186/2197-425X-3-S1-A167

**Published:** 2015-10-01

**Authors:** TA Ayazoğlu, A Baysal, A Özensoy, H Yilmaz, THG Yilmaz

**Affiliations:** Medeniyet University Goztepe Research and Training Hospital, Anesthesiology and Reanimation, Istanbul, Turkey; Kartal Kosuyolu Research and Training Hospital, Anesthesiology and Reanimation, Istanbul, Turkey; Iskenderun State Hospital, Anesthesiology and Reanimation, Hatay, Turkey

## Introduction

Postoperative complications related to pulmonary and cardiovascular functions are higher in patients with chronic obstructive pulmonary disease (COPD) after cardiac surgery (1).

## Objectives

The two different weaning trial methods including T-tube and biphasic positive airway pressure (BiPAP) was compared in patients with COPD after failed extubation postoperatively.

## Methods

After cardiac surgery, 188 patients with COPD under mechanical ventilation for greater than 24 hours and failed extubation were prospectively randomized to receive a T-tube spontaneous breating trial (n = 94) or 2-hour BIPAP trial (n = 92). Two patient who refused to complete two-hour BIPAP was excluded. The trial procedure was repeated after 24 hours if patients were failed to be weaned in the first attempt in case they fulfilled the weaning criteria. Signs of a low 2-hour trial tolerance; included spontaneous respiratory rate >25/min, SatO2 < 90%, FiO2≤0.4, heart rate >140/min (or more than 20% change from the initial heart rate), systolic blood pressure >200 mm Hg or < 80 mm Hg, Pao2≤60 mm Hg, pH≤7.30, and restlessness. ([1]). BIPAP was applied in spontaneous breathing at inspiratory positive airway pressure of 8 to12 cmH2O and expiratory positive airway pressure of 4 to 6 cmH2O. Arterial blood gas values (pH, PaCO2, PaO2, SaO2, HCO3) were collected. The weaning outcome was assessed depending on the following parameters: extubation success, mechanical ventilation duration, duration of intensive care unit (ICU) stay, reintubation rate, and mortality rate.

## Results

Two-hour trial failed in 41 (44%) patients in T-tube and 40 (43%) patients in BIPAP group(p = 0.871). Of patients in whom weaning failed, 33 (54%) in the T-tube group and 29 (73%) in the BIPAP group were successfully extubated (p < 0.001). Mechanical ventilation lasted significantly longer in T tube than in BIPAP group (187 hours vs 163 hours, respectively, p < 0.001). Also, patients in T-tube group ICUstay time was more than patients in BIPAP group (238 hours [interquartile range 208-274] vs 205 hours [200-255], respectively, p < 0.001). Reintubation was required in 9 (27%) and 5 (17%) patients in T-tube and BIPAP group, respectively (p < 0.001) and 30-day mortality was 9(9.6%) and 6 (7%) patients, respectively (p > 0.05).

## Conclusions

After cardiac surgery, patients with COPD who failed the 2-hour spontaneous breathing trial show successful extubation and improved postoperative outcomes as well as diminished ICU stay with the use of BIPAP method rather than T tube method for weaning from mechanical ventilation and for these reasons BIPAP shows a promising weaning modality for mechanically ventilated COPD patients after cardiac surgery.
